# Autocrine VEGF Isoforms Differentially Regulate Endothelial Cell Behavior

**DOI:** 10.3389/fcell.2016.00099

**Published:** 2016-09-21

**Authors:** Hideki Yamamoto, Helene Rundqvist, Cristina Branco, Randall S. Johnson

**Affiliations:** ^1^Department of Physiology, Development and Neuroscience, University of CambridgeCambridge, UK; ^2^Department of Cell and Molecular Biology, Karolinska InstitutetStockholm, Sweden

**Keywords:** endothelial cells, hypoxia, VEGF isoforms, autocrine, endothelial adhesion, intercellular communication, vascular assembly

## Abstract

Vascular endothelial growth factor A (VEGF) is involved in all the essential biology of endothelial cells, from proliferation to vessel function, by mediating intercellular interactions and monolayer integrity. It is expressed as three major alternative spliced variants. In mice, these are VEGF120, VEGF164, and VEGF188, each with different affinities for extracellular matrices and cell surfaces, depending on the inclusion of heparin-binding sites, encoded by exons 6 and 7. To determine the role of each VEGF isoform in endothelial homeostasis, we compared phenotypes of primary endothelial cells isolated from lungs of mice expressing single VEGF isoforms in normoxic and hypoxic conditions. The differential expression and distribution of VEGF isoforms affect endothelial cell functions, such as proliferation, adhesion, migration, and integrity, which are dependent on the stability of and affinity to VEGF receptor 2 (VEGFR2). We found a correlation between autocrine VEGF164 and VEGFR2 stability, which is also associated with increased expression of proteins involved in cell adhesion. Endothelial cells expressing only VEGF188, which localizes to extracellular matrices or cell surfaces, presented a mesenchymal morphology and weakened monolayer integrity. Cells expressing only VEGF120 lacked stable VEGFR2 and dysfunctional downstream processes, rendering the cells unviable. Endothelial cells expressing these different isoforms in isolation also had differing rates of apoptosis, proliferation, and signaling via nitric oxide (NO) synthesis. These data indicate that autocrine signaling of each VEGF isoform has unique functions on endothelial homeostasis and response to hypoxia, due to both distinct VEGF distribution and VEGFR2 stability, which appears to be, at least partly, affected by differential NO production. This study demonstrates that each autocrine VEGF isoform has a distinct effect on downstream functions, namely VEGFR2-regulated endothelial cell homeostasis in normoxia and hypoxia.

## Introduction

Vascular function is intimately connected to endothelial cell function, metabolism, and physiology: vessel integrity is a function of endothelial cell behavior, which in turn affects simple morphological and physiological parameters that impact on the response to organ demand, physiological context, and environmental stress or disease (Ferrara et al., 2003; Coultas et al., 2005). It is thus no surprise that endothelial dysfunction underlies a myriad of pathologies, or occurs as a consequence of unresolved organ failure, such as what is seen in hypertension, diabetes, or atherosclerosis (Carmeliet and Jain, 2000; Forstermann and Munzel, 2006; Eelen et al., 2015).

Vascular Endothelial Growth Factor A (VEGF) is a major regulator of endothelial cell (EC) function, by mediating processes related to survival, proliferation, adhesion and migration, all of which germane to functional and responsive vasculature, and all occurring downstream of receptor activation: VEGF receptor 1 (VEGFR1) is mostly involved with precursor recruitment and intercellular communication, namely with macrophages and monocytes, whereas VEGF receptor 2 (VEGR2) activity underlies endothelial-specific functions (Olsson et al., 2006). VEGF was originally identified as a heparin-binding polypeptide mitogen (Ferrara et al., 1991). Molecular cloning reveals the existence of at least three species of VEGF in mice, which are 120, 164, and 188 amino acids-long. The mitogenic function of VEGF is conditioned by the affinity of carboxyl-terminal domain to heparin (Keyt et al., 1996), a factor that affects the secretion pattern and distribution of individual isoforms. Isoforms that include a heparin-binding domain encoded by exons 6 and 7 are cell-bound, as this domain mediates VEGF binding to heparan sulfate proteoglycan proteins and to cell surfaces (Ferrara et al., 1991; Park et al., 1993; Cohen et al., 1995).

Low oxygen tension up-regulates VEGF in many types of cells through increased stability of VEGF mRNA, as well as through direct transcriptional regulation by hypoxia-inducible transcription factors (Ikeda et al., 1995; Forsythe et al., 1996). It has been asserted that both paracrine and autocrine VEGF signaling is important for vascular modeling, but the autocrine function of VEGF is essential for vascular development and homeostasis (Lee et al., 2007). VEGF Isoforms are generated through alternative splicing (Houck et al., 1991), and can be produced in different ratios, depending on cell type, organ or physiological setting, and varying between very soluble (VEGF120) to membrane-bound (VEGF188), with the most abundant isoform standing somewhere in-between and co-existing in diffusible and bound forms (VEGF164) (Ng et al., 2001). During embryonic development, VEGF188 is most frequently detected in organs that are vascularized through vasculogenesis (e.g., lung, heart, and liver) while the levels of VEGF120 and VEGF164 are more abundant in organs vascularized primarily through angiogenesis (e.g., brain, eye, small intestine, and kidney) (Ng et al., 2001; Haigh, 2008).

More recently, VEGFxxxb isoforms have been described as inhibitory or anti-angiogenic (Bates et al., 2002; Woolard et al., 2009) and overexpressed as regulatory factors for their pro-angiogenic counterparts, for example in involution of infantile hemangioma (Ye et al., 2016) or thought to allow tumor vascularization when downregulated in Renal Cell Carcinoma (Bates et al., 2002). The xxxb variants, formed by distal splicing of exon 8 of the VEGF gene, lack binding sites that allow co-activation of multiple receptors, such as Neuropilin 1. These are neither easily identified, nor appear to be present in most healthy cells and tissues (Harris et al., 2012), which renders their role more relevant in pathologic conditions. For that reason, we did not include a scrutiny of inhibitory xxxb isoforms in this study.

VEGF is required for survival in neonatal mice (Gerber et al., 1999) and also endothelial VEGF null animals are highly compromised during development (Lee et al., 2007). A number of studies have clarified the role of each VEGF isoform during early development by means of organ specific analyses (Carmeliet et al., 1999; Mattot et al., 2002; Ruhrberg et al., 2002; Stalmans et al., 2002; Zelzer et al., 2002; Maes et al., 2004; Darland et al., 2011). Through those analyses, the functional differences between soluble VEGF and matrix-binding VEGF in vascular development have been emphasized. For instance, VEGF164 and/or VEGF188 are critical for myocardial angiogenesis (Carmeliet et al., 1999), whereas VEGF164 is sufficient for the developmental angiogenesis in mouse retina (Stalmans et al., 2002). One of our preceding studies also defined the functional differences and unique functions of each VEGF isoform in tumorigenic neo-vascularization (Grunstein et al., 1999). Additional evidence has shown that single VEGF isoform-producing tumor cells have a distinct biological behavior (Kanthou et al., 2014) and tumors respond differently to anti-angiogenic therapy. Nonetheless, the role of these three VEGF isoforms in autocrine homeostasis of endothelial cells is not yet fully understood.

In this study, we used single VEGF isoform-expressing mice and embryos to isolate primary pulmonary endothelial cells, and investigated the influence of endogenous VEGF isoforms on cell function and response to hypoxic stress. We show that individual VEGF isoforms are uniquely associated with stabilization of VEGFR2 and cell adhesion molecules under hypoxia, and gained insight into the intracellular signaling pathways regulating hypoxia responses and endothelial cell homeostasis downstream of VEGF.

## Materials and methods

### Animals

All animals were housed in a UK Home Office guideline-approved facility, and sample isolation from animals was performed by a license holder in accordance with the UK Home Office regulations under the Animals (Scientific Procedures) Act 1986. The strategies for generating VEGF^120/120^ mice (deletion of exons 6 and 7), VEGF^164/164^ mice (deletion of exon 6), and VEGF^188/188^ mice (containing all exons, except for exon 6b) have been described by other groups previously (Carmeliet et al., 1999; Stalmans et al., 2002).

### Endothelial cell isolations and culture

Mouse endothelial cells were isolated from lungs of VEGF^164/164^, VEGF^188/188^, or littermate wild-type mice at the age of 8–12 weeks old, as previously described (Dong et al., 1997; Tang et al., 2004; Branco-Price et al., 2012). VEGF^120/120^ endothelial cells were isolated from lungs of E17.5 embryos due to the occasional early birth from VEGF^120/+^ female of 18 dpc with the high rate of perinatal deaths of those pups. WT cells from E17.5 were also collected and characterized for proliferation and gene expression. Recombinant mouse VEGF164 protein (R & D Systems, Minneapolis, MN, USA) was added as the supplement for the VEGF^120/120^ endothelial cell culture for at least 7 days after the isolation to rescue the suppressed proliferation. Cells were cultured in endothelial cell growth medium, consisting of low glucose DMEM:F12 with 1% penicillin/streptomycin, 1% nonessential amino acids, 2 mM sodium pyruvate, 20 mM HEPES. Twenty percent fetal bovine serum (FBS) (Gibco) and 75 μg/mL endothelial mitogen (Alfa Aesar, Ward Hill, MA, USA) were added for the complete growth medium.

### Immunofluorescence

The expression and localization of VE-cadherin, β-catenin, and Caspase-3/7 were visualized using immunofluorescence and confocal microscopy (Leica CTR 6500). Cells were cultured on the slide chamber coated with 0.1% gelatin (Sigma-Aldrich), grown to confluence, and serum starved overnight, and then put in hypoxia (1% oxygen, for up to 48 h) or kept in normal oxygen tension for the same length of time. Cells were washed and fixed with 4% paraformaldehyde for 10 min, permeabilized with 0.5% Triton-X100 for 5 min, and blocked with 10% goat serum (Sigma-Aldrich), or 10% donkey serum (Sigma-Aldrich), or 3% bovine serum albumin (BSA) (Life Technologies) in phosphate buffered saline (PBS) for 1 h, and incubated with the following primary antibodies; anti-VE-cadherin (1:50, Santa Cruz Biotechnology), anti- β-catenin (1:50, BD Transduction Laboratories). Alexa Fluor 488- or 568-conjugated secondary antibody (Life Technologies), and FITC-conjugated secondary antibodies (Santa Cruz Biotechnologies) were used at 1:100. Caspase-3/7 Green Flow Cytometry Assay kit (Thermo Fisher) and NucRed 647 (Life technologies) was used for Caspase-3/7 staining and counterstaining of nucleus, according to the manufacture's protocol.

### Cell proliferation assay

Each VEGF isoform cell colony was seeded at density of 2.0 × 10^4^/mL in a 24-well plate with complete culture medium (containing 20% FBS) or reduced serum (containing 2% FBS), or 0.1% BSA containing basal medium. For the analysis of cell proliferation under hypoxia, hypoxic treatment was started 24 h after plating. Data were collected from three wells per each genotype, each isolated from three mice.

### Migration assay

To assess cell migration, wound-healing assay was performed as previously described (Yamamoto et al., 2010). The cells were plated on 10 ng/mL fibronectin (Biomedical technologies)-coated 6-well plates. The monolayer of endothelial cells was scratched manually with a plastic pipette tip. After washing with PBS, the wounded monolayers of cells were allowed to heal for 24 h under hypoxia (1% O_2_) in the serum-free culture medium. The length of the wounds was measured and expressed as the relative folds to the migration distance of the wild type.

### Adhesion assay

Twenty-four-well, non-tissue culture-treated plates were coated with 10 ng/ml fibronectin (Biomedical technologies) for 2 h at 37°C. 1.0 × 10^5^ cells suspended in 200 μL serum free media were added to each well and incubated at 37°C for 30 min. The supernatant was removed and adherent cells were selected by washing with PBS. Three wells per group were incubated for 5 min with 0.1% crystal violet in 20% methanol and then washed three times with PBS. Crystal violet contained in the adhesive cells was extracted in 50% ethanol and quantified by the absorbance at 590 nm.

### Permeability assay

Endothelial cells were seeded at the density of 1 × 10^5^ cells into COSTAR transwell (6.5 mm diameter, 0.4 μm pore, Corning, cat no. 3413). Cells were grown for 4 days and then pre-incubated at 21 or 1% O_2_ for 3 h. HRP-conjugated streptavidin (1 ×, Abcam) was then added to the top of the upper chamber; after 1 h, 100 μL of medium was collected from the lower chamber, and the level of HRP-streptavidin in the lower chamber was measured by mixing the 100 μL medium with equal volume of 3,3′,5,5′-tetramethylbenzidine (TMB) peroxidase enzyme immunoassay substrate (Abcam), incubated for 30 min before the addition of 50 μL of Stop Solution (Abcam). The absorbance of the solution was measured at 450 nm.

### Transendothelial migration assay

Transendothelial migration of GFP-labeled Lewis Lung Carcinoma cells (LLC^GFP^) was analyzed, as previously described (Branco-Price et al., 2012) with some modifications. Endothelial cells were seeded on COSTAR transwell inserts (6.5 mm diameter, 8 μm pore; Corning, cat no. 3422) at cell density of 2.0 × 10^4^ per well, and grown for 5 days. LLC^GFP^ cells, pre-stained with Calcein-AM dye (BD Bioscience), were seeded on the endothelial monolayer and the inserts were incubated at 37°C for 24 h under hypoxia (1% O_2_), with serum-free media on both upper and lower chambers. Migrated cells were counted in five random fields per insert at 100x magnification by using the fluorescent stereomicroscope (Leica M165FC).

### Tube formation assay

*In vitro* tube formation on Matrigel was analyzed, as previously described (Tang et al., 2004), with some modifications: Growth Factor Reduced Matrigel (BD Biosciences) was applied at 60 μL/well in 96-well plates and incubated at 37°C for 30 min to allow hardening. 6.0 × 10^3^ primary lung endothelial cells medium containing 0.5% serum, were seeded on top of the Matrigel. Plates were incubated under normoxia or hypoxia (1% O_2_) at 37°C for 9 h. Cells were stained with Calcein AM dye (BD Bioscience) at the end of the incubation, and parameters of detected networks were analyzed using Image J software (Angiogenesis Analyzer, created by Gilles Carpentier).

### Quantification of NO levels

Culture medium collected from the primary endothelial cells at the time point of 48 h under hypoxia at 1% oxygen or under normoxia were analyzed using an NOA280i (Siever, GE Healthcare) according to the manufacturer's instructions. Readings were performed a minimum of three times for each of three wells.

### Collection of extracellular matrix fraction

Extracellular matrix was prepared from a culture dish, as previously described (Yamamoto et al., 2009) with the following modifications: Total cell lysates in 100 mm dishes had been collected in 500 μL RIPA buffer [10 mM Tris/HCl pH 8.0, 150 mM NaCl, 5 mM EDTA, 0.5% Sodium deoxycholate, 0.1% sodium dodecyl sulfate (SDS), 1% TritonX-100] supplemented with protease inhibitors (Roche). Extracellular matrix remaining on the dish was extracted at 100°C for 5 min in 375 μL of LDS Sample buffer (1x, Invitrogen) after washing with PBS and RIPA buffer three times.

### Semi-quantitative qPCR

Total RNA was extracted using RNeasy Mini Kit (QIAGEN) and converted into cDNA from 0.5 μg or 1 μg of total RNA using Superscript III (Invitrogen) according to the manufacturer's protocol. cDNA was amplified in SYBR Green with an ABI Prism system (Applied Biosystems). Forward and reverse primers were as follows: integrin alpha V 5′-AGGCTGGAACT CAACTGCTC-3′, 5′-TTGGCCCGTC AATGTCGTAA-3′; integrin β 3 5′-GCCTTCGTG GACAAGCCTGT-3′, 5′-GGACAATGC CTGCCAGCCTT-3′; β-actin 5′-CCCAGAGCA AGAGAGG-3′, 5′-GTCCAGACGCAG GATG-3′. Results were normalized to β-actin mRNA levels.

### Reagents and antibodies

1400 W and LY294002 were purchased from Sigma-Aldrich and Cell Signaling Technology, respectively. VEGF Mouse Elisa kit (Abcam, cat no. ab100751) was used for the quantitative analysis of VEGF in culture medium. Anti-VEGFR2 (D5B1, Cell Signaling Technology, 9698) antibody and Protein A/G agarose (Santa Cruz Biotechnology, sc-2003) were used for VEGFR2 immunoprecipitation. The detail of primary antibodies used for western blot or immunofluorescence analyses are as following; VEGF (P-20, Santa Cruz Biotechnologies, sc-1836), VEGFR2 (D5B1, Cell Signaling Technology, 9698), VE-cadherin (Santa Cruz Biotechnology, sc-6456), β-actin (A5316, Sigma-Aldrich), α-phosphotyrosine (4G10, Millipore, 05-1050), phospho-AKT (Ser 473, Cell Signaling Technology, 9271), phospho-AKT (Thr 308, Cell Signaling Technology, 13038), β-catenin (BD Transduction Laboratories, 610153), PECAM-1 (M-20, Santa Cruz Biotechnology, sc-1506), ICAM-1 (M-19, Santa Cruz Biotechnology, sc-1511), VCAM-1 (Abcam, ab174279).

### Statistical analyses

Each experiment was performed isolating from at least two different animals per group and three technical replicates. The statistical significance was assessed by Student's *t*-test and expressed as average ± SEM. *P* < 0.05 was accepted as significant.

## Results

### VEGF isoforms differentially regulate endothelial cell proliferation and viability under hypoxia

**Figure 1:**
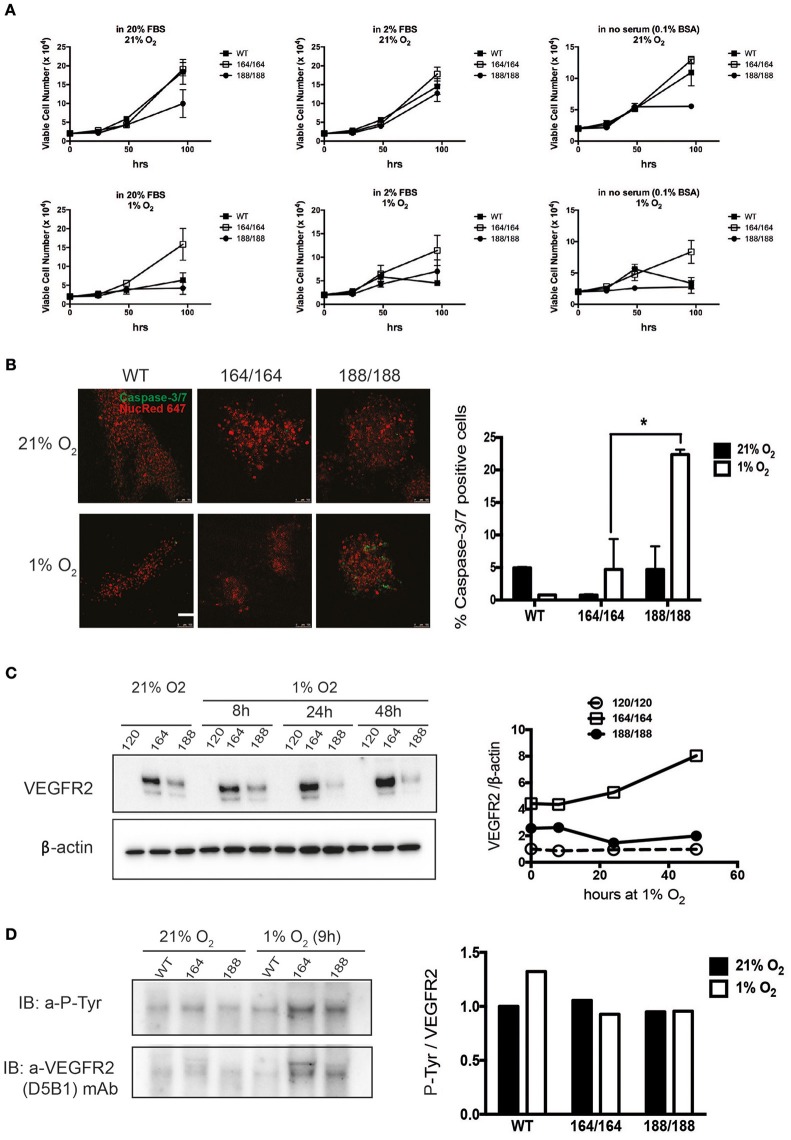
**Contribution of specific VEGF isoforms to endothelial cell viability and autocrine signaling. (A)** Endothelial cell viability was measured in wild-type (WT) endothelial cells and cells expressing each of the VEGF isoforms 164 or 188, in normoxia (21% O_2_, top panels) and hypoxia (1% O_2_, bottom panels), for 96 h. The culture media containing 20% FBS (high serum), 2% FBS (low serum), or 0.1% BSA (no serum) were replaced at the time points of 24 h after cell plating. Data shown are average cell number (per well) ± SEM (*n* = 3); three wells, each containing cells from 2 to 3 mice. **(B)** EC of different genotypes were stained for Caspase 3/7 to assess relative levels of apoptosis in normoxia (top) and after 48 h of hypoxia (bottom), scale bar, 100 μm. Right Panel shows ratio of caspase-3/7-positive cells out of total cells (caspase-3/7-positive and NucRed Live 647-positive cells). Data represents average ± SEM (*n* = 3); three fields, each containing cells from 2 animals, ^*^P < 0.05. **(C)** VEGFR2 protein abundance was assessed by western blot of whole cell extracts (left). Quantification is shown in the panel on the right. **(D)** Activation of VEGFR2 was assessed by immunodetection of P-Tyr in total VEGFR2 protein obtained by immunoprecipitation (IP); representative blot shown on the left and quantification data on the right.

To investigate the differential roles of endogenous VEGF isoforms on endothelial cell (EC) viability under hypoxic stress, we purified ECs from mice expressing exclusively one of the isoforms. We started by monitoring the proliferation rates of cells expressing single isoforms, and our first observation was that EC expressing single VEGF120 (VEGF120 EC) displayed severely suppressed proliferation in all tested conditions (Figure 1A), for which reason it was not included in these panels. Characterization of growth was therefore carried out solely on VEGF164 EC and VEGF 188 EC, and cell proliferation was quantified over a range of serum concentrations. We see that autocrine expression of VEGF164 conferred a significant proliferative advantage; the maximal difference between VEGF164 EC and other cells was noted at 96 h of hypoxic culture (Figure 1A).

The proliferation of VEGF188 EC was stalled after 48 h without serum (Figure 1A, far right panels). Another measure of viability can be the rate of spontaneous apoptosis, which was visualized by Caspase-3/7 staining, and demonstrates that VEGF188 EC are more apoptotic than VEGF164 EC, and that this difference is exacerbated by hypoxia, where there is a five-fold higher frequency of apoptotic cells in VEGF188 than in VEGF164 EC populations (Figure 1B). This is consistent with previous knowledge that the mitogenic activity of VEGF relies on its ability to effectively bind heparin (Keyt et al., 1996; Ferrara, 2010) and, consequently, cell surfaces and receptors, and explains why EC expressing exclusively VEGF120, which exists only as a soluble and diffusible signal (Supplementary Figure 1) do not proliferate and are only partially sustained in culture with supplementation of a potent mitogenic isoform, VEGF164.

### Vascular endothelial growth factor receptor 2 stability in hypoxia is differentially affected by individual VEGF isoforms

VEGF exerts its effects by binding to multiple receptor tyrosine kinases, VEGF receptor 1, 2, and 3 (VEGFR1-3), and co-receptors such as Neuropilins 1 and 2. VEGFR1 is a regulator of VEGFR2 signaling capacity, whereas VEGFR3 is associated with lymphangiogenesis by VEGFC and D (Olsson et al., 2006). VEGFR2 is implicated in all aspects of normal and pathological vascular endothelial-cell biology, and its expression is restricted to endothelial cells (Olsson et al., 2006). It has also been shown that VEGFR2 expression increases in response to hypoxia via a hypoxia-inducible factor (HIF)-1α-driven, VEGF-mediated autocrine loop on EC (Tang et al., 2004). Protein levels of VEGFR2 in EC under normoxia were evaluated by western blot analysis of total cell lysates of EC isolated from each genotype, as shown in Figure 1C. We used fresh isolates of VEGF120 EC to investigate the levels and activation of VEGFR2 in these cells, and strikingly, EC expressing exclusively VEGF120 were also unable to stabilize VEGFR2, in agreement with their failure to divide. WT EC purified from E17.5 littermates proliferated normally (not shown) and expressed normal levels of VEGFR2 (Supplementary Figure 2). VEGF164 is found in conditioned medium and cell lysates, and VEGF188 almost exclusively bound to extracellular matrix (Supplementary Figures 1, 3). VEGFR2 is most abundant in normoxic VEGF164 EC, which is also the only genotype where hypoxia-dependent induction of VEGFR2 is observed. On the other hand, tyrosine phosphorylation of VEGFR2 (normalized to total VEGFR2 levels) was equivalent in both VEGF164 EC and VEGF188 EC under hypoxia (Figure 1D). This is an indication that autocrine VEGF164, but not VEGF188, is necessary for the hypoxia-induced up-regulation of VEGFR2, whereas the phosphorylation of VEGFR2 under hypoxia is not necessarily correlated with VEGFR2 levels. We do see an increase in P-VEGFR2 in WT cells in response to hypoxia, suggesting that both VEGF164 and VEGF188 are required for the increased activation levels of this receptor in response to hypoxia.

### Hypoxic endothelial cells expressing each VEGF isoform differ in morphology and barrier function

To investigate the distinct autocrine roles of each VEGF isoform on endothelial barrier function, morphology and expression levels of cell junctional architecture was assessed and compared in each culture. At endothelial adherens junctions, VEGFR2 coexists with β-catenin and vascular endothelial (VE)-cadherin, known to regulate the half-life and the internalization of VEGFR2 (Calera et al., 2004; Lampugnani et al., 2006). VEGF signaling itself also induces the endocytosis of VE-cadherin (Gavard and Gutkind, 2006). We see the highest levels of VE-cadherin in VEGF164 EC during the 48-h course of hypoxia, and the lowest in VEGF120 EC (Supplementary Figure 4). β-catenin levels, also induced by hypoxia, were elevated in both VEGF164 EC and VEGF188 EC, and maintained at high levels throughout the hypoxic treatment, whereas the induction of this protein in WT EC is transient, decreasing after the 8 h time point. Lowest levels of this protein were found in VEGF120 EC (Supplementary Figure 4).

**Figure 2:**
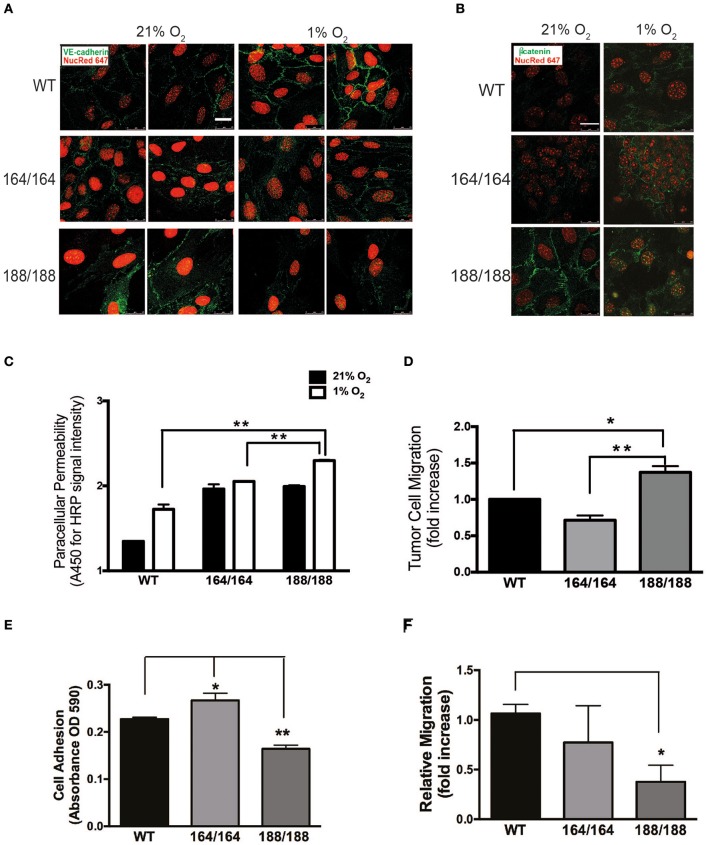
**Endothelial integrity as a function of isoform-specific VEGF signaling. (A)** Normoxic and hypoxic (1% O_2_, 24 h) EC were fixed and immunostained with VE-cadherin and **(B)** β-catenin antibodies and detected by immunofluorescence. Targets are shown in green, nuclei in red; scale bars, 25 μm. **(C)** EC monolayers were grown in 0.4 μm pore Boyden chamber filters. To assess paracellular permeability, normal medium was replaced with medium containing HRP-streptavidin (~100 kDa), which was quantified in the lower chamber 1 h after normoxic or hypoxic incubations using a peroxidase-coupled assay; data shown are absorbance values at 450 nm. **(D)** Tumor cell invasion assay: GFP-labeled Lewis Lung Carcinoma cells (LLC^GFP^) were seeded over endothelial monolayers in 8 μm pore filters; fluorescent cells were counted on the opposite side of the filter after 24 h at 1% O_2_. Results shown in **(C)** and **(D)** are average ± SEM (*n* = 3); three wells isolated from three mice per each group. **(E)** A suspension of 1.0 × 10^5^ endothelial cells was seeded onto fibronectin-coated plates and washed with PBS 30 min later. Adherent cells were stained with Crystal Violet, which was subsequently extracted and quantified by spectrometry. Results shown are average ± SEM (*n* = 3); three wells, each containing cells isolated from 2 to 4 mice. **(F)** Scratch assay performed on endothelial cell monolayers, and cell migration was quantified 24 h after monolayer disruption by measuring scratch width after 24 h in at 1% O_2_. Results shown are average ± SEM (*n* = 4); four fields, each of cells isolated from 2 to 4 mice. ^*^P < 0.05, ^**^P < 0.005.

The morphology of EC expressing individual VEGF isoforms was investigated by immunofluorescent (IF) staining of VE-cadherin and β-catenin (Figures 2A,B). VEGF164 EC and WT EC formed a cohesive and uniform monolayer. In contrast, VEGF188 EC displayed a more mesenchymal appearance with elongated, spindle-shaped cells with extended processes, correlated with little cell-cell association. Furthermore, VE-cadherin of VEGF188 EC was ectopically internalized instead of being localized to the cell surfaces (Figure 2A). In hypoxia, only WT EC showed increased signal for VE-cadherin, implying that hypoxia induction of VE-cadherin levels or increased network formation may require the concomitant expression of all VEGF isoforms (Figure 2A). β-Catenin IF also showed close longitudinal alignment along EC in VEGF164 EC, localizing to the cell membrane. Instead, in VEGF188 EC, β-catenin was seen in the nucleus (Figure 2B). In hypoxia, cytosolic β-catenin levels were increased in all EC lines, including WT (data not shown).

The hypoxia-driven increase in endothelial permeability was more pronounced in VEGF188 EC than in VEGF164 EC (Figure 2C), with WT EC showing the lowest paracellular permeability, an indication that all three VEGF isoforms are required for effective EC barrier function. PECAM-1, classically involved in several types of EC adhesion junctions, was most abundant in VEGF164 EC (Supplementary Figure 4). Similarly, VEGF188 EC monolayers were more permissive to tumor cell migration (LLC^GFP^) than VEGF164 EC monolayers (Figure 2D).

### Endothelial VEGF164 and VEGF188 are differentially necessary for hypoxia-induced tube formation

We next investigated angiogenic potential in each type of EC by performing adhesion, migration, and network formation assays. Previous studies, which analyzed vascular development and angiogenesis of mouse embryos expressing only VEGF120, underscored the importance of matrix-binding isoforms, VEGF164 and/or VEGF188, for cardiovascular development (Carmeliet et al., 1999; Ruhrberg et al., 2002; Van Den Akker et al., 2007, 2008). The adhesion of EC toward the extracellular matrix protein fibronectin was significantly higher in VEGF164 EC and lower in VEGF188 EC, when compared to that of WT (Figure 2E). In scratch assays on fibronectin-coated plates, hypoxic VEGF188 EC showed decreased cell migration relative to WT and VEGF164 EC (Figure 2F). Integrin β3 subunit, a component of the binding structure to fibronectin, was up-regulated by hypoxia in VEGF164 EC (Supplementary Figure 5), suggesting that VEGF164 is more critically involved in cell adhesion and migration of EC than VEGF188.

**Figure 3:**
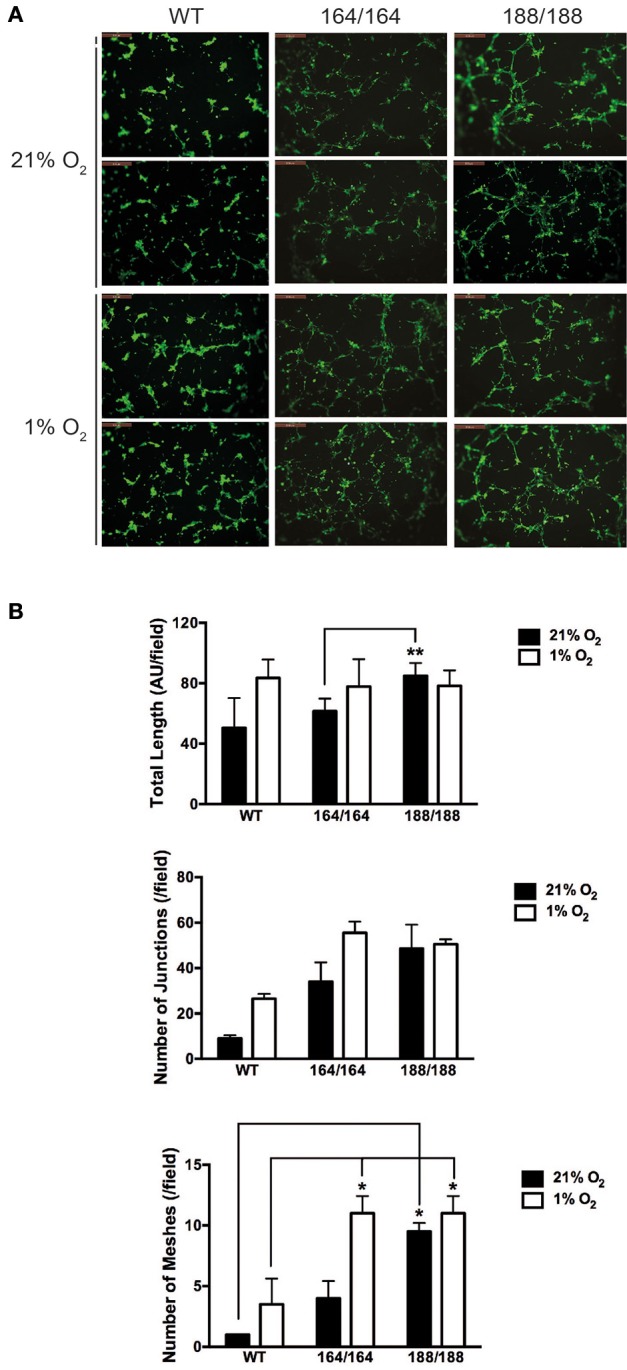
**VEGF isoform contribution to endothelial network formation. (A)** Tube formation assay on growth factor-reduced Matrigel. Cells were incubated for 9 h in 21% O_2_ or 1% O_2_ and resulting networks were visualized with Calcein AM live cell staining. Scale bars, 500 μm. **(B)** Quantification of different parameters of endothelial networks by Image J software, shown as average ± SEM (*n* = 3); three fields, each group of cells obtained from 2 to 3 mice. ^*^P < 0.05, ^**^P < 0.005.

To determine the role of specific isoforms in angiogenesis, each EC genotype was plated onto Growth Factor Reduced Matrigel and allowed to self-organize into capillary-like structures. The basal angiogenic potential in normoxia was higher in VEGF188 EC than in VEGF164 EC (Figures 3A,B), with VEGF188 EC extending with more frequent junctions and mesh-like structures in normoxia than VEGF164 EC (Figures 3A,B). In contrast, formation of capillary-like mesh structures, induced by low oxygen tension, was profound in VEGF164 EC but not in VEGF188 EC (Figures 3A,B), suggesting the differential importance of VEGF164 and VEGF188 on network maturation under hypoxia and normoxia, respectively, due to distinct molecular characteristics.

### VEGF188, but not VEGF164, is associated with hypoxia-induced nitric oxide production via PI3K/Akt pathway

**Figure 4:**
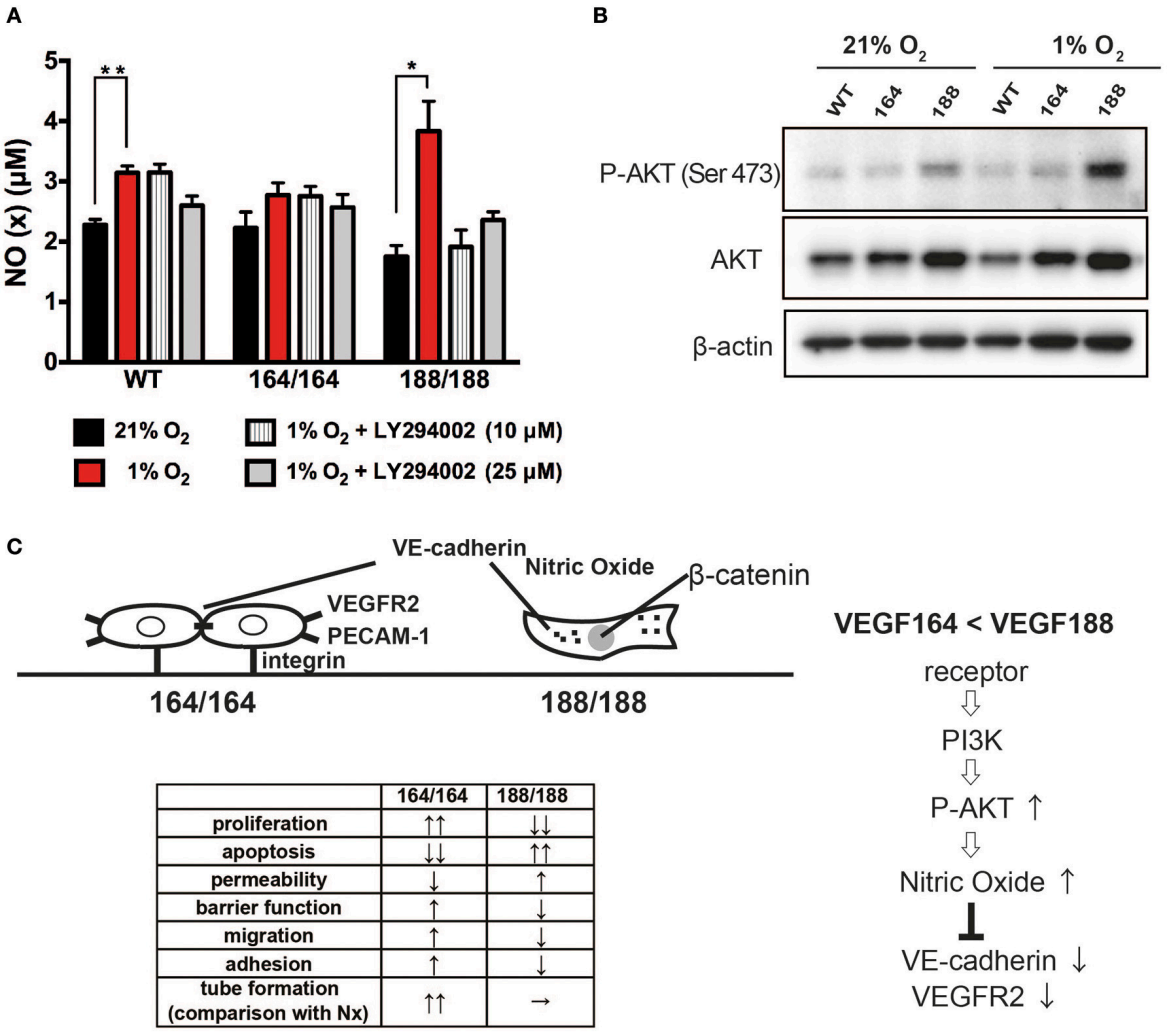
**VEGF isoform contribution to nitric oxide generation by endothelial cells through PI3 kinase/Akt signaling during hypoxia. (A)** NO (x) levels in cell conditioned medium of EC exposed to 21 or 1% O_2_ for 24 h, with or without 1 h of LY294002 pre-treatment at the indicated concentration. Data shown are average ± SEM (*n* = 3); three wells, each containing cells isolated from 2 to 3 mice. ^*^P < 0.05, ^**^P < 0.005. **(B)** Western blot of whole cell extracts from endothelial cells treated for 24 h at 21 or 1% O_2_ were probed with phospho-Akt at Ser473, total Akt, or β-actin antibodies. **(C)** Schematic representation of plausible differential signaling in endothelial cells exclusively expressing VEGF164 or VEGF188.

Nitric Oxide (NO) is a bioactive modulator of various types of cells leading to the regulation of vascular tone, immune response, and synaptic neurotransmission (Napoli et al., 2013). It is known that endogenously produced NO can increase endothelial cell permeability, and suppress cell proliferation through cell cycle arrest of EC (Tanner et al., 2000; Villalobo, 2006). Endothelial NO synthase (eNOS) is a fundamental determinant for physiological NO production in EC. eNOS is activated by the serine/threonine protein kinase Akt/PKB and is up-regulated by VEGF (Bouloumie et al., 1999; Dimmeler et al., 1999). We have shown that NO production by EC in hypoxia is significantly increased by activation of the inducible NOS isoform (iNOS), a target of HIF-1α (Branco-Price et al., 2012). To investigate the role of VEGF isoforms in hypoxia-induced NO production by EC, we measured accumulation of NO in EC-conditioned media. NO was detected in all types of EC under normoxia, and the hypoxia-induced NO production was significant only in VEGF188 EC and WT EC, whereas VEGF164 EC did not show any increments of NO by hypoxia (Figure 4A). Treatment with LY294002, an inhibitor of phosphatidylinositol-3-OH kinase (PI3K), attenuated the hypoxia-induced NO release from VEGF188 EC and WT EC, but had no effect on the NO production by VEGF164 cells (Figure 4A), indicating the possible relationship between hypoxia-induced NO production of EC and activated PI3K under autocrine VEGF188. As the downstream molecules of PI3K pathway, hypoxia-induced phosphorylation of Akt at Ser473 was highest in VEGF188 EC (Figure 4B). Phosphorylation of Akt at Thr308 was not promoted by hypoxia (data not shown). Treatment with 1400 W, an iNOS-specific inhibitor, cumulatively increased hypoxia-mediated VEGFR2 stability (Supplementary Figure 6). These data indicate that each VEGF isoform acts distinctly on hypoxia-dependent stabilization of VEGFR2, and the underlying process is, at least partially, a consequence of differential NO production, as a result of altered PI3K/Akt activation.

## Discussion

In this this paper we focused on the role of isoforms VEGF120, 164 and 188 in vascular endothelium homeostasis, which includes their response to hypoxia: in these cells, the ability to sense, withstand and respond to a wide range of oxygen concentrations is key to their ability to perform functions and rescue organs and tissues during insults such as altitude, vascular occlusion, or hypertension (Tretyakov and Farber, 1995; Ten and Pinsky, 2002); the endothelial response underlying the vascular remodeling are what allow subsequent tissue endurance. We hypothesized that the plasticity of endothelial metabolism includes the control of the ratio of the VEGF isoforms in the endothelium, such as what was shown in lung epithelium during pulmonary injury (Medford et al., 2009).

While characterizing the distribution of VEGF isoforms, we showed that all isoforms are induced by hypoxia: VEGF120, the smallest and most diffusible isoform, as expected, is only present in conditioned media (Supplementary Figure 1), whereas VEGF164 is found in all fractions, confirming that this isoform the most ubiquitous, and found in diffusible, cell- and matrix-bound, whereas VEGF188 appears mostly in the extracellular matrix (Supplementary Figures 1, 3).

What appears to be the most striking effect of this differential distribution is the autocrine activation of VEGFR2, the main receptor involved in EC function. VEGF is known to cause VEGFR2 accumulation and activation (Lee et al., 2007), as seen in the typical hypoxia response (Tang et al., 2004), but VEGF120 alone is unable to drive this process, and even significantly increased levels of this isoform, such as observed during hypoxia exposure (Supplementary Figure 1), are not enough to promote VEGFR2 stabilization, resulting in unviable homozygous offspring in VEGF120 animals, and in inability to maintain proliferation of cells in culture, even with supplementation. Conversely, VEGF164 EC displayed constitutively high levels of VEGFR2, which increased with increased exposure to hypoxia (Figure 1C), concomitant with increased activation by tyrosine phosphorylation (Figure 1D). Interestingly, VEGF188 has moderate levels of VEGFR2, which do not increase with hypoxia, but this does not compromise VEGFR2 phosphorylation: both VEGF164 and VEGF188 have identical ratio of activated VEGFR2 which is not altered by hypoxia, a phenomenon only observed in EC expressing all isoforms (WT) (Figures 1C,D). This argues that the differential accumulation of VEGFR2 protein relies profoundly on VEGF164, whereas phosphorylation happens downstream of either isoform, with the increase of VEGFR2 phosphorylation in response to hypoxia likely requiring the concomitant expression of both, or even all, isoforms, as suggested by the increase in P-VEGFR2/VEGFR2 ratio in WT EC.

Functions downstream of VEGFR2 activation include survival and proliferation, which, as expected, are higher in VEGF164 and WT cells, whereas VEGF188 EC exhibit lower and unsustained proliferation and increased apoptosis (Figures 1A,B).

Subsequent functional analyses confirmed VEGF164 to be the isoform driving stability of endothelial monolayers, with increased adhesion to matrices and EC-EC communication, with significantly higher VE-cadherin levels correlating with decreased paracellular permeability and, conversely, increased barrier function, to both solutes and invading cells (Figures 2, 3, Supplementary Figure 4). VEGF188 EC showed almost opposite phenotypes, except in angiogenic potential, as both EC isotypes are able to form networks (Figure 3). However, VEGF188 EC have increased tube formation in normoxia. This can be explained by the less adhesive nature of these cells, which should render them more likely to move and assemble. The fact that there is no increase in tube formation in VEGF188 EC in response to hypoxia, could be due to the fact that this isoform alone is not sufficient to drive increase in VEGFR2 levels and activation (Figures 1C,D).

Finally, it has long been established that endothelial cells are key regulators of vascular relaxation (by the production of NO) (Vanhoutte, 1989; Cines et al., 1998), and interestingly, are also the cells that produce the most potent vasoconstriction signal (endothelin). Most vascular pathologies are associated with impaired vascular relaxation, which potentiates shear stress and accumulation of reactive oxygen species (which in turn compromise NO synthesis) (Cines et al., 1998; Forstermann and Munzel, 2006). NO homeostasis is a result of eNOS activity, whereas iNOS can be activated in EC during physiological insult, such as hypoxia (Branco-Price et al., 2012; Forstermann and Sessa, 2012). We investigated if NO production happens downstream of VEGF signaling, and saw significantly higher levels of NO in VEGF188 cells, when compared to WT and VEGF164 concomitant with increased P-Akt levels, known to promote eNOS activity. Changes in P-Akt levels can shift the homeostatic setpoint, and a biased ratio of VEGF188 can lead to a more relaxed endothelium (Figure 4C).

It is implausible that VEGF120 has an autocrine role in EC, and, as proposed in one of ours and others previous studies, this isoform acts instead as a paracrine signal, which can create a gradient and direct recruitment of other cells, including endothelial cells from preexisting vessels (Grunstein et al., 2000; Ruhrberg et al., 2002; Gerhardt et al., 2003). This is corroborated by a significant increase in secretion of VEGF120 in hypoxia (Supplementary Figure 1), a signal that could drive vessel sprouting and guidance toward oxygen deprived sites, such as during injury, inflammation, tumorigenesis, or regeneration.

Taken together (Figure 4C) these data suggest that VEGF164 and VEGF188 are the isoforms with autocrine function in EC, and likely act in coordination to maintain homeostasis. By altering the ratio of VEGF164 to VEGF188, the endothelium can respond to physiological demand by improving barrier function (more VEGF164), increasing permeability (more VEGF188), or assemble new vessels. This is relevant in physiological and in clinical settings. Isoform-promiscuous approaches to treatment can now be refined to address this more intricate level of control of vascular function.

Our future work includes assessing the role of these isoforms in other endothelial cell types, to identify endothelial-intrinsic and organ-driven regulation of endothelial function, in health and disease, as well as development.

## Author contributions

HY acquired and analyzed all the data, and all authors contributed to experimental design and interpretation of the work. HY and CB wrote the manuscript, which was critically revised by HR and RSJ. All authors agreed to publishable version of this article and are equally accountable for all the aspects of the work presented.

## Funding

This work was funded by Wellcome Trust PRF to RSJ.

### Conflict of interest statement

The authors declare that the research was conducted in the absence of any commercial or financial relationships that could be construed as a potential conflict of interest.
