# Supplementary material for: Autocrine VEGF Isoforms Differentially Regulate Endothelial Cell Behavior
Source: Front Cell Dev Biol. 2016 Sep 21;4:99. doi: 10.3389/fcell.2016.00099 (PMC5030275; doi:10.3389/fcell.2016.00099)
Supplement: Supplementary file 1 [file DataSheet1.PDF]

Supplementary Material

Autocrine VEGF isoforms differentially regulate endothelial cell behavior

Hideki Yamamoto[a], Helene Rundqvist[b], Cristina Branco[a, *], and Randall S. Johnson[a, b, *]*

Correspondence: Randall S. Johnson: rsj33@cam.ac.uk, Cristina Branco: cmm77@cam.ac.uk

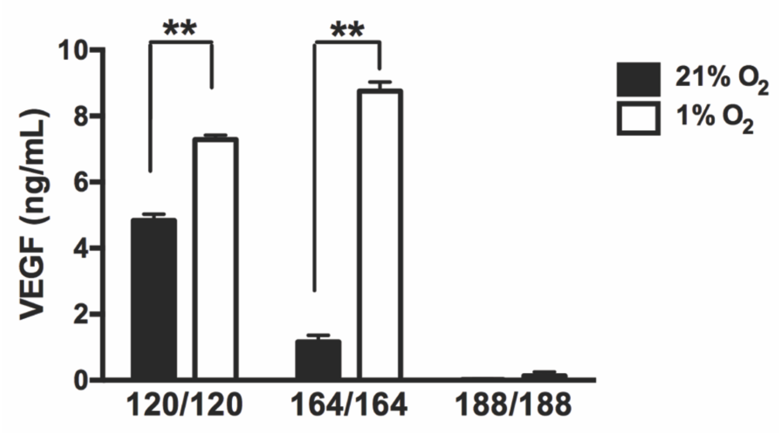

Supplementary Figure 1. Levels of secreted VEGF were analysed by ELISA of isoform-specific EC conditioned media, after normoxia (21% O2) or hypoxia (1% O2) for 48 h. ** *P* < 0.005.

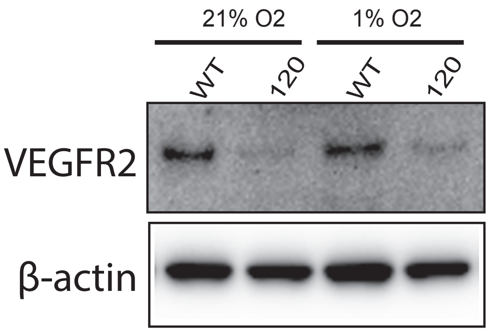

Supplementary Figure 2. Protein levels of VEGFR2 in endothelial cells from lungs of E17.5 embryos expressing wild type VEGF or single VEGF120. Western blot of whole extracts from each type of cells exposed to 21% O2 or 1% O2 for 24 h was probed with VEGFR2 and β-actin antibodies.

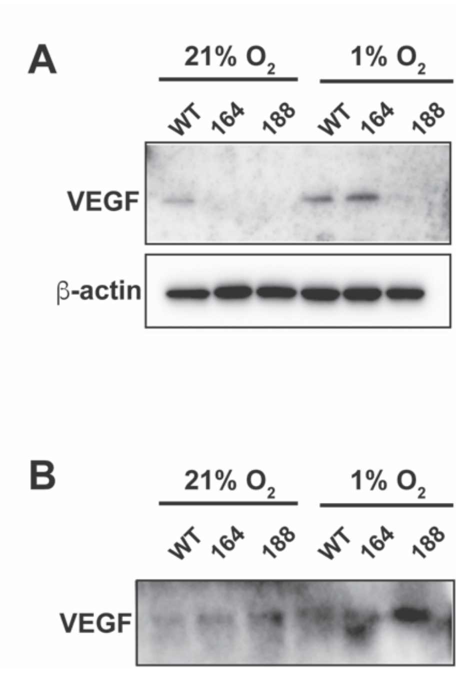

Supplementary Figure 3. Distribution of each VEGF isoform expression in EC cultured in normoxia (21% O_2) or hypoxia (1% O_2) for 48 h. **(A)** Retention of VEGF in the whole cell lysates of endothelial cells expressing single VEGF164, single VEGF188, or wild type VEGF. **(B)** Western blot of extracellular matrix fraction prepared from endothelial cells expressing single VEGF164, single VEGF188, or wild type VEGF was probed with VEGF antibody.

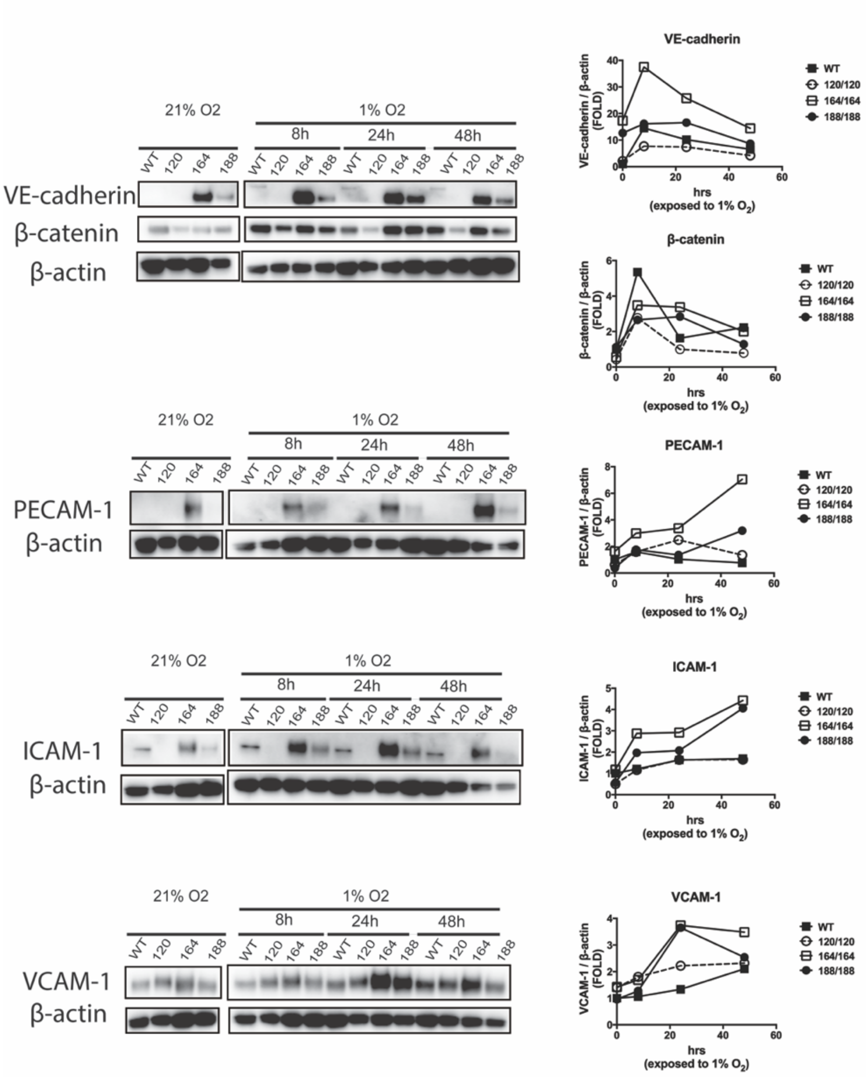

Supplementary Figure 4. Protein levels of adhesion molecules VE-cadherin, β-catenin, PECAM-1, ICAM-1, and VCAM-1 were assessed by western blot and quantified in endothelial cells expressing single VEGF isoforms or wild-type VEGF, exposed to 1% O_2 for up to 48 h.

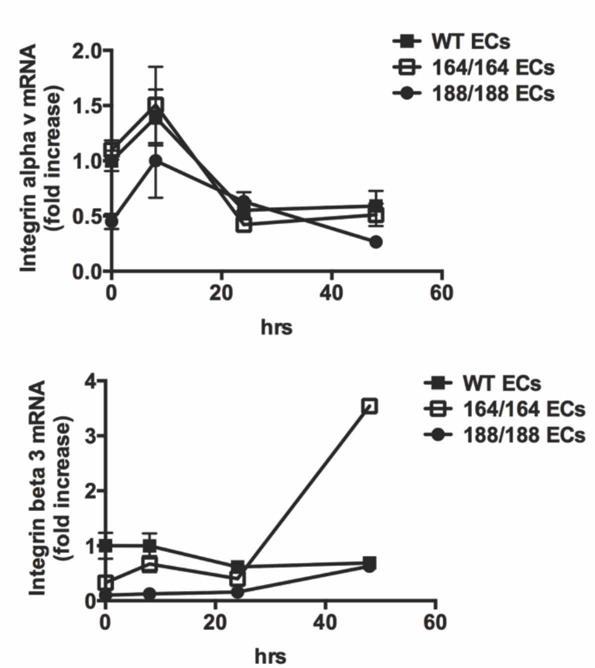

Supplementary Figure 5. mRNA from endothelial cells, exposed to 1% O_2 for up to 48 h was used to quantify steady state levels of Integrins αv and β3 (components of the receptor for fibronectin) by qPCR . Data were shown as average ± SEM (n = 3).

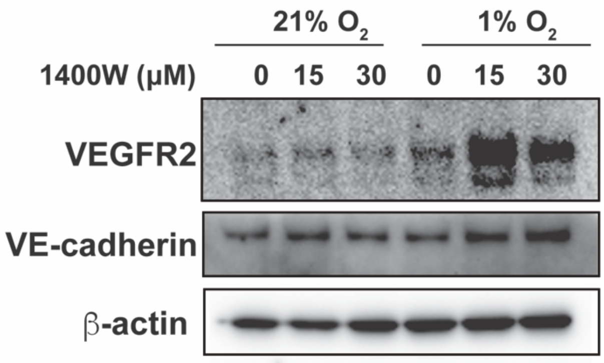

Supplementary Figure 6. iNOS inhibition effects on the stability of VEGFR2 and VE-cadherin of endothelial cells, exposed to normoxia (21% O_2) or hypoxia (1% O_2) for 24 h.
